# Unification of Road Scene Segmentation Strategies Using Multistream Data and Latent Space Attention

**DOI:** 10.3390/s23177355

**Published:** 2023-08-23

**Authors:** August J. Naudé, Herman C. Myburgh

**Affiliations:** Department of Electrical, Electronic and Computer Engineering, University of Pretoria, Pretoria 0002, South Africa

**Keywords:** scene segmentation, self-driving, dual attention mechanisms, road scene understanding, data fusion

## Abstract

Road scene understanding, as a field of research, has attracted increasing attention in recent years. The development of road scene understanding capabilities that are applicable to real-world road scenarios has seen numerous complications. This has largely been due to the cost and complexity of achieving human-level scene understanding, at which successful segmentation of road scene elements can be achieved with a mean intersection over union score close to 1.0. There is a need for more of a unified approach to road scene segmentation for use in self-driving systems. Previous works have demonstrated how deep learning methods can be combined to improve the segmentation and perception performance of road scene understanding systems. This paper proposes a novel segmentation system that uses fully connected networks, attention mechanisms, and multiple-input data stream fusion to improve segmentation performance. Results show comparable performance compared to previous works, with a mean intersection over union of 87.4% on the Cityscapes dataset.

## 1. Introduction

Road scene understanding, as a field of research, has attracted increasing attention in recent years due to advancements in technology that provide hardware and software that are increasingly capable of executing resource-intensive tasks and because such systems are easy to install for various applications, resulting in improvements in accessibility, performance, and affordability. Another factor contributing to the technological and market push for better advanced driver assistance systems (ADAS) and advanced driving systems (ADS) has been the motivation to significantly improve road safety. It has been observed that distracted driving behaviour can significantly increase the chances of accidents, with a direct correlation with road accident statistics [1,2].

However, the development of road scene understanding capabilities has seen numerous complications as reported in various works [3,4,5,6]. This has largely been due to the cost and complexity of achieving human-level scene understanding. These systems may take many different forms and inspirations from other fields of engineering and science, but ultrasonic, RADAR, LiDAR, and camera sensors have been commonly utilised to solve the challenges of road scene understanding [7,8,9].

Almost all road scene understanding systems share a fundamental initial task that has been deemed as crucial for performing accurate road scene analysis. This is called the perception step and pertains to the ability of a system to sense or perceive its environment of operation [10]. Therefore, road scene understanding systems must be reliable and consistent, failing which unnecessary safety risks might be introduced by these systems, which would defeat the purpose of such implementations, i.e., dangerous driving decisions, undetected road scene hazards, or failure to comply with road laws and regulations.

Some factors affecting perception accuracy, reliability and scalability of ADS and ADAS are often attributed to environmental factors. These systems frequently operate under highly dynamic conditions such, as at different times of day and under varying weather conditions, illumination angles, and especially traffic conditions. Computer-vision-based systems are notorious for becoming very unreliable in adverse weather conditions. LiDAR specifically struggles when utilised in snowy driving scenarios, where too much snow may obscure certain road features from the LiDAR sensor [7,8,11]. This is due to the high reflectivity of snow. Camera-based ADS and ADAS have seen a surge in popularity, mostly due to new techniques and methodologies that allow such implementations to negate some disadvantages of other sensor hardware. This indicates that road scene understanding systems are becoming cheaper to implement, as in some instances, cameras can replace very expensive LiDAR equipment. Market trends have also contributed, with the continual success of automotive tech companies such as Tesla and Comma AI.

Popular approaches reported in previous works can be categorised into a few high-level groups: (i) object recognition and localisation, (ii) segmentation, and (iii) end-to-end learning. Some studies have even opted to combine certain aspects of these approaches. Object recognition refers to a classification process that strives to classify or group images or features into distinct categories. Depending on the use case, object recognition can be used to classify road-scene-specific objects for various uses. It is well known that not all elements of a driving scene require the same degree of attention. Therefore, by classifying different entities in a scene, they can each be processed accordingly and much more efficiently—compared to processing everything in a scene the same manner. Object localisation has often been added to recognition pipelines and refers to the process of locating features in the image space relative to other features in the same space [12]. This has provided ADAS and ADS with the ability to not only sense object types but also where they are in relation to other objects [13]. Improved object avoidance, path planning, and object prioritisation are some of the most notable improvements introduced by these systems [13,14,15]. Classical object detection systems have utilised sliding window techniques [16].

This paper proposes a more unified road scene segmentation system that utilises a combined approach to road scene segmentation. Several segmentation techniques and related works are discussed in this paper, and the proposed system is described. This system strives to improve road scene understanding by combining these techniques. Specifically, multi-input stream data fusion, trainable upscaling, and a dual attention module are combined with a fully connected network (FCN) backbone architecture. A custom loss function is also implemented to optimise for mean intersection over union (mIoU), which is a common segmentation metric used in related works.

## 2. Related Works

Various studies have emphasised the benefits of breaking the scene analysis problem into smaller subsystems that each focus on different feature types [12,17]. Some studies have proposed the utilisation of stereoscopic imagery to create depth image information via the use of occupancy disparity maps. This allows for such systems to perform obstacle detection based on generated depth information [9,13]. Similar to many other road scene analysis systems, these systems also utilise information obtained from various pieces of sensor hardware. They create occupancy disparity maps that, when overlaid with the original RGB images, can provide probabilities of how far away various objects are from the vehicle [9].

Computer-vision-based systems have often been compared to human vision, since how humans perceive different road scenes and traffic scenarios can be considered one of our best benchmarks with which to compare the performance of these systems. Humans have an innate ability to process specific visual cues very efficiently and very effectively. However, Computer-vision-based systems often have to infer the same information from video streams and other low-level sensors. This has led to the identification of the semantic gap that was proposed in [18]. The semantic gap was derived from the problem of autonomous vehicles having to process spatial, spectral, and temporal information at a sufficient level. As a result classification, systems are impacted more by this matter, as finer classifications are required. However, various studies have proposed methods to overcome this problem by using variations of data fusion [13,17,18,19].

Scene segmentation is a form of classification implemented on a per-pixel level [20]. This classification technique has allowed various implementations to extract higher-level features from different driving scenes [18,21,22], meaning that each pixel in a given image space was classified into road-scene-specific classes.

Various scene segmentation techniques have been proposed, and some perform better than others when applied to road scene analysis applications. Some have utilised colour space information (RGB) captured from road scene images. Colour-based implementations typically perform mathematical preprocessing on the colour space of input images, such as progressive image smoothing. Tarel and Bigorgne [23] proposed a growing algorithm that takes certain regions of the image space that are known to be road surface regions as initialisation seeds.

Scene segmentation has been shown to be possible using RGB images, edge maps, and texture-based information. These methods often suffer from unreliable performance when used in real-world scenarios [18,24,25]. Deep learning, in particular, has been involved in extensive contributions in this research space [3,7,8,20,21,22,26,27,28,29].

The creation of a more unified ADS or ADAS has been described by some as being a very difficult problem [17]. However, a few studies have proposed unification strategies that have shown promise in terms of building more unified road scene understanding systems [4,18,28,30,31]. One study, in particular, managed to merge two basic input types using a fully connected network (FCN) backbone architecture. The *s-FCN-loc* system was proposed in [27]. This proposed system utilises both RGB colour images and channel-duplicated contour images. Not only does the *s-FCN-loc* system utilise a unique parameter-sharing concept, but it also includes the use of a pregenerated location prior map [27], allowing the system to minimise computational complexity and to learn combined features from all channel elements. Contour images are created from the RGB images using a pretrained model based on structured forests [27].

In pursuit of even more unified ADS and ADAS, several studies have proposed “black-box” systems that utilise end-to-end learning [20,31,32,33,34]. End-to-end learning often refers to the process of learning a direct mapping between input vectors and end-goal output vectors [33]. These systems often have direct driving control processing added to their processing pipelines, meaning that they can achieve some degree of autonomous driving directly based on raw camera inputs. However, some authors have noted that further research is required to improve and better measure the robustness of such a convolutional neural network (CNN) system. The main difference between end-to-end learning systems and other scene understanding methods is required training techniques. Behavioural cloning requires a strict balance between wanted and unwanted training samples.

End-to-end learning models can be implemented in a very efficient manner, but what they lack in computational complexity, they make up for in training complexity. Due to the nature of these systems, they often act like black-box models, which makes monitoring their training parameters very difficult. Most performance monitoring can only be done on the input and output sides of these models, meaning that researchers cannot easily peek inside the model to better observe how it finds mappings between inputs and outputs. Overall model performance can be increased by scaling up to larger and deeper models, but this would also correspond to an immense increase in the number of required training samples. It would also make calculating how the model works at various stages computationally expensive [5,20,31,32,33,34]. All of the factors mentioned above result in a model that is computationally cheap to run but very expensive to train.

Although most of the sources mentioned thus far proposed road scene understanding techniques and methodologies that can be categorised into broad groups, some have proposed techniques that differ considerably from conventional techniques in these research areas [35]. Han et al. proposed two systems that based on generative adversarial networks (GANs) [35]. These systems function on the basis that one network generates data samples and another network classifies those samples in terms of whether they belong to a specific subset. From a high-level perspective, GANs operate using competing algorithmic functions, where one network learns to create better fake samples, and the other network, in turn, learns to better distinguish fakes from legitimate samples. This results in a weakly supervised training approach in which models can be trained with less manual intervention and labelling [35]. The main motivation for this approach is to lessen the inherent dependencies of FCN models on strict, manually labelled datasets.

Another source took a less conventional approach to object detection and classification using a 3D CNN architecture. The utilisation of stereoscopic depth images and RGB colour images allowed researchers [36] to create volumetric point cloud representations of objects. Each pixel was projected into a 3D matrix space, referred to as a volumetric pixel or voxel. The main disadvantage of an approach such as this was found to be the efficiency and increased computational complexity of processing 3D CNN models. Although this approach may be beneficial for obstacle detection, it might hinder overall ADAS or ADS performance. Therefore, this method was deemed unsuitable for more unified road scene understanding systems.

As previously discussed, road scene understanding systems frequently utilise some form of computer vision or machine learning approach, implying a heavy dependency on large training data and training algorithms. However, numerous sources have investigated improved training techniques [6,21,22,37,38,39]. The author of various studies have noted that their results may have been negatively impacted by not having access to adequate datasets. When such systems are not trained on an adequate number of training samples, they often fail to infer critical features from smaller dataset, resulting in scene understanding models that do not perform optimally or perform incorrectly in some cases. However, among studies that opted to use existing datasets, some were found to be more popular for road scene understanding than others. Some of the more popular datasets are the Cityscapes dataset [21], the KITTI dataset [39], and the CamVid dataset [40].

Following this trend, other sources have also proposed various synthetic dataset creation techniques in which simulators or virtual renderings are used to create semirealistic driving scenarios and environments. Simulators such as Cognata deep learning autonomous simulation, Udacity self-driving car sim, Microsoft AirSim, and CARLA are popular dataset creation tools in road scene understanding research [41,42,43,44]. Not only have these tools provided more efficient ground truth generation, but they provided also given researchers with much more control over the driving scene.

## 3. Materials and Methods

This section describes the design details, materials, and methods for the unified road scene segmentation system proposed in this study. This system consists of several design aspects that have been used in related works. These design aspects include an FCN backbone network, dual attention modules for latent space attention, trainable upscaling using fractionally strided convolution blocks, skip connections for detailed feature retention, structured forests for efficient edge detection, stereoscopic depth detection, and multi-input data fusion. These design aspects, which are combined in a novel manner, contribute to a more unified road scene segmentation system, achieving competitive road scene segmentation performance, as presented in Section 4.

By utilising multiple input data types (RGB, contour, and stereoscopic depth), the proposed segmentation system may learn more complex features as a result of considering multimodal input data. Utilising an FCN backbone network, the encoder section of the backbone encodes the input data streams into latent space features. A dual attention mechanism then learns contextual focus from these latent space features, allowing the system to capture features in the latent space that would otherwise be outside of the scope of the receptive field of FCN kernels. The spatial attention module captures spatial features based on their locality on each latent feature map, while the channel attention module captures features between latent feature maps. The sum of the attention features is then passed to the decoder section of the backbone for upscaling and recomposition into a segmented road scene image. Skip connections offer detail retention and minimise the vanishing gradient effect that occurs as a result of feature downscaling operations. Explanations of each of these system design aspects are presented in subsequent sections of this paper, with more detail on their implementation and application.

### 3.1. FCN Backbone Architecture

**Figure 1:**
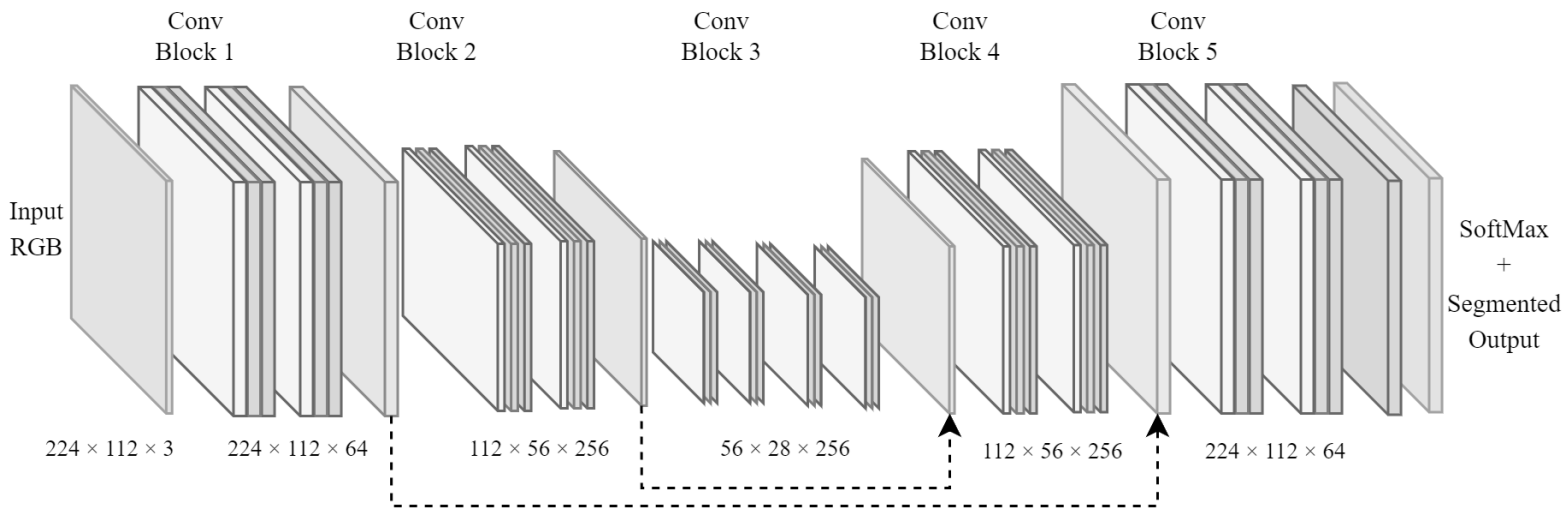
FCN architecture that was chosen as the backbone for the implemented road scene segmentation system.

An FCN architecture was chosen as the backbone of the road scene segmentation system because of how popular such architectures have been in similar systems, providing the ability to process various image sizes much more efficiently by condensing feature maps into lower-dimensional states. Such architectures perform the bulk of their processing before scaling each feature map back up to a specified dimensionality. This also means that they have the ability to output images larger or smaller than the original input images. However, additional learning effort is required for autoencoders to learn how to accurately scale images to extreme sizes. Figure 1 illustrates the backbone FCN architecture that was used in the design of the proposed road scene segmentation system.

The depiction of the FCN architecture in Figure 1 clearly shows the encoder and decoder segments of the network. The encoder portion encodes various features into deeper channel-wise feature maps as pooling layers (at the origin of the dotted arrows) condense spatial dimensions. However, the decoder portion utilises fractionally strided convolutional layers, which iteratively expand the condensed feature maps into their original image resolution. The output of this architecture has dimensions of H×W×N, where *H*, *W*, and *N* are the height, width, and number of classes, respectively. This provides the decoder section with a trainable way to upscale feature maps, which is similar to how trainable pooling layers are achieved.

### 3.2. Skip Connections

The FCN backbone architecture illustrated in Figure 1 also demonstrates the persistence of skip connections between layers. These connections are denoted by arrows pointing from specific sections in the architecture to others to show the flow of information through the network. The use of skip connections in the network was inspired by multiple sources, such as [31,45,46], which emphasise the need to preserve spatial information from earlier layers in the architecture.

Pooling operations are largely to blame for the vanishing gradient effects observed in many deep learning models. They downscale layer outputs to smaller sizes, and in doing so, they lose some information. In some research fields, such as computer vision, higher-resolution imagery has often led to greater performance gains from deep learning models, although without entirely compensating for the negative effects of pooling operations.

### 3.3. Additional Input Data Streams

The road scene segmentation system developed for this study was adapted to utilise multiple input data streams. The concept of multiple input data sources for deep learning models has been covered in several sources. The main inspiration to add this feature for road scene segmentation is presented in [27]. It was observed from various sources that some of the most difficult regions in a scene to segment are the boundaries between two segmentation zones. These regions consist of pixel data that show transitional information from one object class to another, and sometimes three or more classes meet at these junction points of image space. In these specific cases, the ground truth labels comprise those pixels assigned to their correct classes. However, because the edge pixels are so close to other class labels, deep learning models often struggle to place them in their correct class groupings. The aforementioned skip connections actively help to improve deep learning performance in this regard. Another effective approach to improve boundary performance was proposed in [27], i.e., the use of contour data (sometimes referred to as edge data).

**Figure 2:**
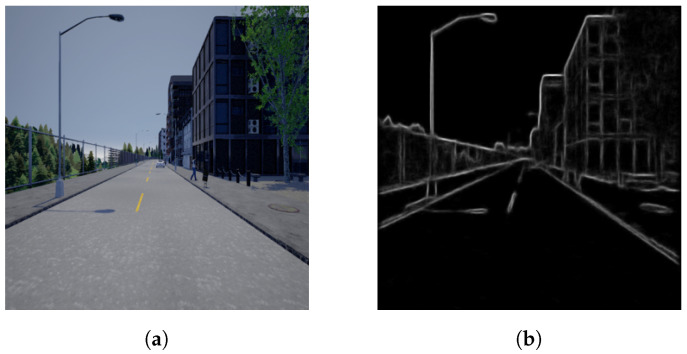
Contour map representation created using a pretrained structured forest model and equivalent RGB input source. (**a**) RGB input image. (**b**) Converted contour map.

The proposed system utilised a pretrained structured forest model to generate contour images from a given RGB image, similar to the model used in [27], and was found to be effective at highlighting outer boundaries instead of textured edges found within objects, as illustrated in Figure 2.

The use of structured forests for fast and efficient edge detection in the *s-FCN-loc* system was inspired by [47]. The process of converting RGB or RGB-D images into semantic contour maps—similar to that shown in Figure 2—functions by utilising random decision trees. However, because these random decision trees are complex to train in this fashion, the authors opted to aggregate several decision trees into an ensemble model (structured forests).

Depth map images is another input data type implemented in the proposed system, meaning that the system utilises a total of three input data streams alongside the aforementioned RGB images and contour maps. Depth map images can be created from stereoscopic camera systems that combine two captured perspectives and produce an image with contextual depth information. This data type can often be produced alongside standard RGB images, with data typically referred to as RGB-D images. However, for this implementation, the choice was made to process depth data separately in a different network pipeline. The main inspiration to include this data type came from [48,49], both of which showed that by utilising contextual depth information, road scene segmentation performance can be improved.

**Figure 3:**
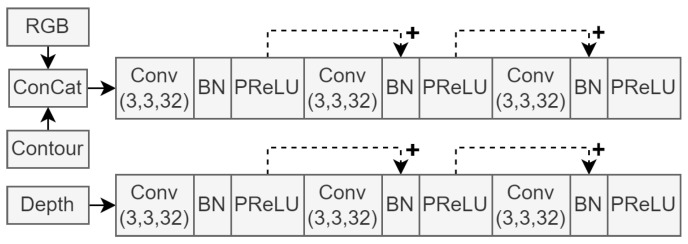
First three convolution blocks, illustrating the input data streams of the network architecture.

Figure 3 shows the input sections of the proposed system architecture, where each of the aforementioned data types is represented as input to its independent convolutional pipeline. It is worth noting that the output from each batch normalisation (BN) operation was summed with the skip connections, as illustrated by dashed arrows. The resulting output feature maps of this input module were fed to the decoder segments in the network as skip connections. They were also fed to the second stage of the encoder.

Figure 3 shows that the RGB and contour images are concatenated before entering a separate processing line from the depth images. This implementation decision was inspired by the *s-FCN-loc* system reported in [27]. Sharing a processing line of convolutional layers provides a form of weight sharing between network input images and allows elements of one data type to influence the feature extraction process of the other. Since the information contents of RGB and contour data are similar in nature, these data types were grouped together in this way. Depth data do not contain colour or object boundary information. Instead, they convey information about object distances, already representing a higher-dimensional data type. Therefore, we decided to include decided a dedicated processing line of convolutional layers. Additionally, grouping two or more of the input data streams improves model efficiency, as large-scale convolution blocks are not necessary for each input.

**Figure 4:**
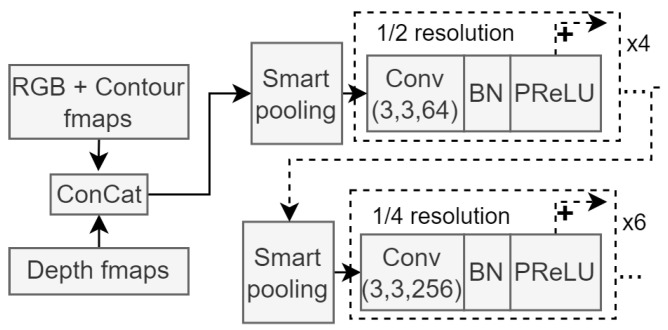
Module architecture for the second stage of the encoder network. Smart pooling refers to the previously discussed trainable downscaling method, and resolution sizes are clearly illustrated. Similar to the input module shown in Figure 3, each skip connection connects to the next repeating BN block.

The following stage of the encoder architecture consists of two pooling segments, which reduce the dimensionality of the feature maps and contain more convolutional blocks, as seen in the input module. The purpose of this module in the network architecture is to extract high-dimensional features and condense the resolution of the feature maps to more manageable sizes. Each pooling operation is followed by four or six convolution blocks. Figure 4 shows this section of the network architecture.

The feature maps from each smart pooling block in Figure 4 are also fed to the decoder module of the segmentation network, retaining fine-grained information when the decoder module upscales the segmentation maps created from deep features.

### 3.4. Attention Modules

Self-attention modules have been around for a while, but they have seen an increase in popularity for use in computer vision in recent years [50]. These modules are good at extracting deep contextual features, and they are good at handling long-range dependencies. The authors of [50] proposed a dual attention network that utilises two attention modules in parallel. This idea was adopted and combined with previous approaches to create an improved road scene segmentation system. Subsequent attention modules operate on the basis of feature maps from the encoder network modules but further downscaled to 1/8 resolution.

#### 3.4.1. Spatial Attention Module

**Figure 5:**
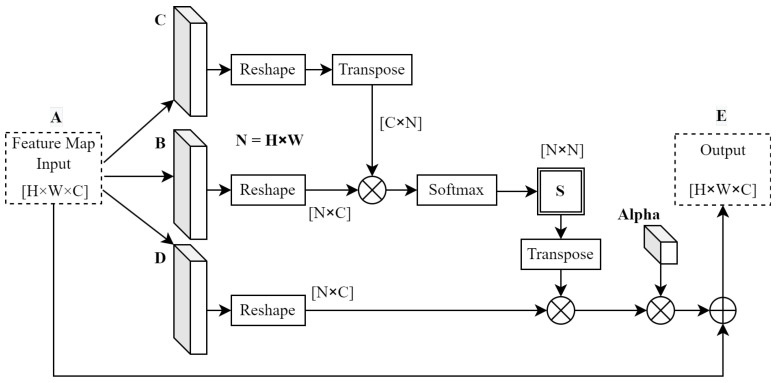
Spatial attention module computation flow. Adapted from [50], © 2019 IEEE.

The spatial attention module creates a contextual mapping between any two pixels in the feature map space. Therefore, it allows data outside the receptive fields of convolution layers to influence the classification labels of those within. The computational flow of this module is represented in Figure 5.

To obtain position features, the attention module shown in Figure 5 passes the input feature maps through 3 independent convolutional layers labelled B, C, and D. This takes advantage of the receptive fields of these convolution operations. Next, each of the resulting feature maps is reshaped, and matrix multiplication is applied to create a position map similar that presented in [50]. A softmax activation is then applied to this position map to create partial classification labels (S) at each position in this matrix. The softmax activation is calculated as

(1) $$s_{ji}=\frac{e^{\left(B_{i}C_{j}\right)}}{\sum_{i=1}^{N}e^{\left(B_{i}C_{j}\right)}}$$

for each element of S; the values are functions of matrices B and C. An intermediate matrix was generated from the matrix multiplication between convolution D and the activated position map (S) so as to apply these activations to the rest of the model. Finally, the output feature map (E) was generated after multiplying a trainable parameter (**α**) by the intermediate matrix, followed by an element-wise summation between the original input feature map and the intermediate matrix. This resulted in the output of this module being a weighted sum of features, where **α** is made trainable to weigh features accordingly [50]. This is calculated as

(2) $$E_{j}=\alpha \sum_{i=1}^{N}\left(s_{ji}D_{i}\right)+A_{j}$$

It is worth noting that the generated position map adopts dimensions of *N* × *N*, where *N* is the product of the height and width dimensions of the original input feature map. The depth dimension was processed in parallel using the channel attention module.

#### 3.4.2. Channel Attention Module

**Figure 6:**
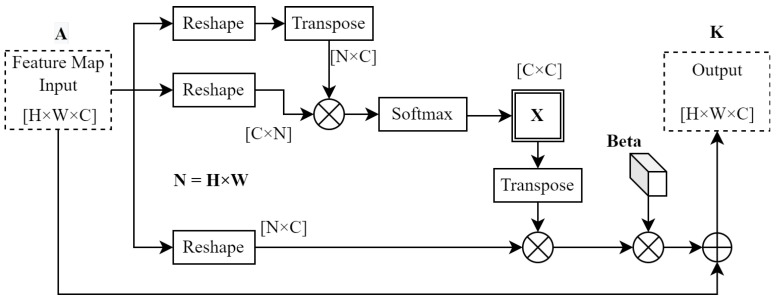
Channel attention module computation flow. Adapted from [50], © 2019 IEEE.

The channel attention module used in this system was also obtained from [50]. The spatial attention module computes positional context between features, but the channel attention module creates a contextual mapping between different filters or feature channels. The computational flow for this module is shown in Figure 6.

Both attention modules compute their output feature maps similarly, with some differences closer to the input side of each module. Whereas the spatial attention module passes the module input through a set of convolution layers, the channel attention module does not in order to conserve inter-feature relationships in the depth dimension [50]. The second difference is with respect to the calculation of matrix X in Figure 6. X can be computed as follows

(3) $$x_{ji}=\frac{e^{\left(A_{i}A_{j}\right)}}{\sum_{i=1}^{N}e^{\left(A_{i}A_{j}\right)}}$$

The softmax operation for X is the same as for S, as shown in Figure 5. However, to calculate X, the softmax only works with matrix A and **$A^{T}$**, resulting in a channel map with dimensions of *C* × *C*. The output of the channel attention module is calculated as

(4) $$K_{j}=\beta \sum_{i=1}^{N}\left(x_{ji}A_{i}\right)+A_{j}$$

Similarities with (2) should be noted. Again, a trainable parameter (β) was implemented to ensure that the output K was the weighted sum of channel features.

### 3.5. Decoder and Auxiliary Decoder Modules

**Figure 7:**
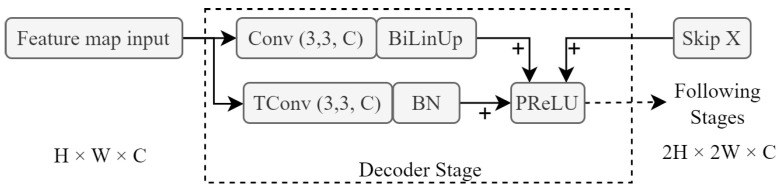
Single-decoder module block that utilises both fractionally strided convolution (TConv) and bilinear upsampling (BiLinUp). Skip X represents the incoming skip connection from earlier modules in the system architecture, and H×W×C represent the dimensions of the feature maps.

In this implementation, the decoder section of the FCN backbone consisted of three stages. Each stage doubles the resolution it receives and uses a fractionally strided convolution layer to do so. After every convolution layer, a PReLU layer is utilised as the activation function. This gives this module the capacity to find learnable scaling solutions instead of relying on non-trainable bilinear upscaling. Figure 7 illustrates the scaling layers for each decoder block.

**Figure 8:**
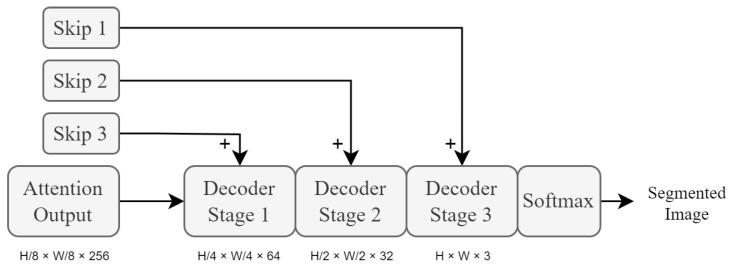
High-level overview of decoder module stages. Each stage uses fractionally strided convolutional layers to upscale the input feature maps at that stage. These layers are then succeeded by PReLU activation layers. H×W×C represent the dimensions of the feature maps.

**Figure 9:**
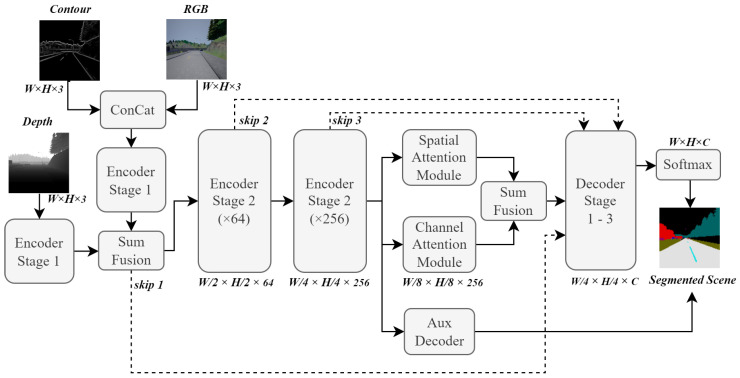
High-level overview of the final road scene segmentation system architecture. H×W×C represent the dimensions of the feature maps.

The proposed network architecture utilises three of these stages, as shown in Figure 8. The final module used in the proposed system is the auxiliary decoder module (depicted as Aux Decoder in Figure 9). This module is a simplified version of the main decoder module in order to conserve computational complexity. It serves no purpose other than to provide an additional auxiliary loss calculation for the training of the system. Not only does an auxiliary loss aid in counteracting the effects of vanishing gradients, but it also creates additional complexity for the encoder modules to process. This provides a form of training focus to the network when the encoder modules are being trained, which improves the network’s ability to perform segmentation-rich feature extraction, as well as training stability.

### 3.6. Final System Overview

An overview of the final road scene segmentation system is shown in Figure 9. This is a high-level depiction of how each component from previous sections is connected to form the final system architecture.

### 3.7. Model Training

Several iterations of the proposed road scene segmentation system were implemented and tested. This was done to ensure adequate understanding of the underlying concepts and to ensure that each design parameter was understood in practice as well as in theory. Due to the expensive nature of deep learning models, especially CNN-based architectures, that process image data, powerful hardware was required to effectively train these models. Input image dimensions and model hyperparameters were chosen to fit within available computational resources.

The development machine had the following specifications and was used as the primary training machine for this study (equipment was sourced in the city of Pretoria, South Africa):

- CPU: Intel Core i7 8700K;
- GPU: ASUS ROG STRIX OC 11 GB RTX 2080 Ti;
- RAM: 32 GB Corsair Vengeance 3000 MHz memory;
- Motherboard: MSI Z370 GAMING PRO CARBON AC;
- PSU: Templarius 850 W;
- Storage: 480 GB Kingston solid-state drive.

The proposed road scene segmentation system was implemented using the Python language, along with various other Python library modules, among which Tensorflow and Keras were used extensively throughout this study. The following module versions were used for this development environment.

- Python 3.7;
- PyCharm Community Edition IDE;
- Tensorflow 2.5.0;
- Keras 2.5.0;
- OpenCV 4.5.5.62;
- Pillow 9.0.1;
- Matplotlib 3.5.1;
- NVIDIA Graphics Driver 516.94;
- NVIDIA GPU Computing Toolkit 11.0;
- NVIDIA cuDNN 11.0.

### 3.8. Dataset Creation and Preprocessing

For this implementation, it was decided to use the CARLA simulator for data collection purposes and to create a custom dataset with which to compare Cityscapes performance results to those reported in [44]. This allowed for much finer control over environmental conditions in driving scenarios. The final dataset contained nearly 13,000 training samples (13 classes). Each sample is accompanied by pixel-accurate ground truth annotations autogenerated by the simulator. The proposed road scene segmentation system was implemented and tested under clear daytime driving conditions only, as adverse weather condition testing was beyond the scope of this research. This dataset was manually created purely from the described simulation environment. It does not include any image samples from the Cityscapes dataset, as it is a secondary dataset separate from the CARLA simulator dataset. Both were independently used to train their own version of the proposed segmentation system, allowing comparisons to made in Section 4.

Each sample and its corresponding depth and ground truth images were scaled to various resolutions in order to test the effects that resolution scaling had on various performance aspects of the proposed system. Before training was initiated for each iteration, the dataset was randomly shuffled, and an 80/20 split was implemented for training and validation samples, respectively. RGB samples were used to generate contour maps before training because this was a simple way to speed up the whole training process. Ground truth images had to be converted into one-hot encoded matrices to train the model. Finally, the dataset was standardised to achieve zero mean and unity variance across the training data samples. For this study, model inputs were set to a resolution of 224 × 112. This resolution was experimentally estimated to be large enough to test the viability of different models but also small enough to not run out of system memory.

It was also observed that not all road-scene-specific classes occur in every scene, causing unbalanced class distributions. To compensate for this, class weighting and label smoothing were implemented during the training process of the proposed system.

### 3.9. Training Cost and Evaluation

The final operation in the proposed system architecture was a softmax layer that classified each pixel in each output feature map into one of the 13 predetermined object classes (20 for the Cityscapes dataset). It is widely accepted by various sources that the most popular cost function for multiclass classification problems is the use of categorical cross-entropy loss. Cross entropy with softmax substitution is calculated as

(5) $$H=-\log(\frac{e^{s_{p}}}{\sum_{j}^{C}e^{s_{j}}})$$

where *H* is the cross entropy, $s_{p}$ is the feature map value, *C* is the represented classes in the classification distribution, and $s_{j}$ is the classification obtained by the softmax operation [51]. Equation (5) is a simplified expression that is commonly referred to as softmax loss, as it already contains terms imposed by the softmax operation before the cross-entropy loss calculation [51,52]. The motivation for using this specific loss function follows the close relationship between cross entropy and the Kullback–Leibler (KL) divergence theorem, which states that a certain probability distribution may be expected from any two separate probability distributions over the same random variable. This allows the uniqueness to be calculated between these two distributions; when one distribution is replaced with that of the ground truth label set, the predicted distributions can be assessed [52].

A second loss function was also investigated. This loss function works by directly minimising the intersection over union (IoU) error generated as a result of mismatching class masks. Referred to as IoU-loss in this study, this cost function computes the overlap error between ground truth and predicted pixel regions. This loss function more directly improved the segmentation capabilities of the proposed models, since it provides error correction for the exact metric that needs to be optimised. IoU-loss is not yet a very well-known or frequently used cost function for segmentation systems. However, it has been gaining popularity as simple way to boost segmentation performance over cross entropy. Cross-entropy-based cost functions have a tendency to focus on the classification of each individual pixel into their respective class. However, IoU loss provides more flexibility for edge pixels that reside on the border regions between multiple other classes, allowing a small number of edge pixels to be incorrectly classified in exchange for allowing fewer errors to occur inside a class mask where no other classes are present. This is often a much more desirable outcome in road scene segmentation systems in which a tradeoff between these two situations needs to be made. IoU can be calculated as

(6) $$\mathrm{IoU}=(\frac{Y_{t}Y_{p}}{Y_{t}+Y_{p}-\left(Y_{t}Y_{p}\right)})$$

where $Y_{t}$ represents the true labels, and $Y_{p}$ represents the predicted labels. This calculates the global IoU for all classes. The error function is simply normalised to $IoU_{loss}=1-IoU$. Finally, the cost function that was utilised to train the final versions of the road scene segmentation model used both functions, and a weighting between them was experimentally obtained. The final loss function is expressed as

(7) $$L=1-(\frac{Y_{t}Y_{p}}{Y_{t}+Y_{p}-\left(Y_{t}Y_{p}\right)})-0.1\log(\frac{e^{s_{p}}}{\sum_{j}^{C}e^{s_{j}}})$$

## 4. Results

In this section, the proposed road scene segmentation system is evaluated using the Cityscapes dataset and the CARLA dataset.

### 4.1. Model Performance

**Figure 10:**
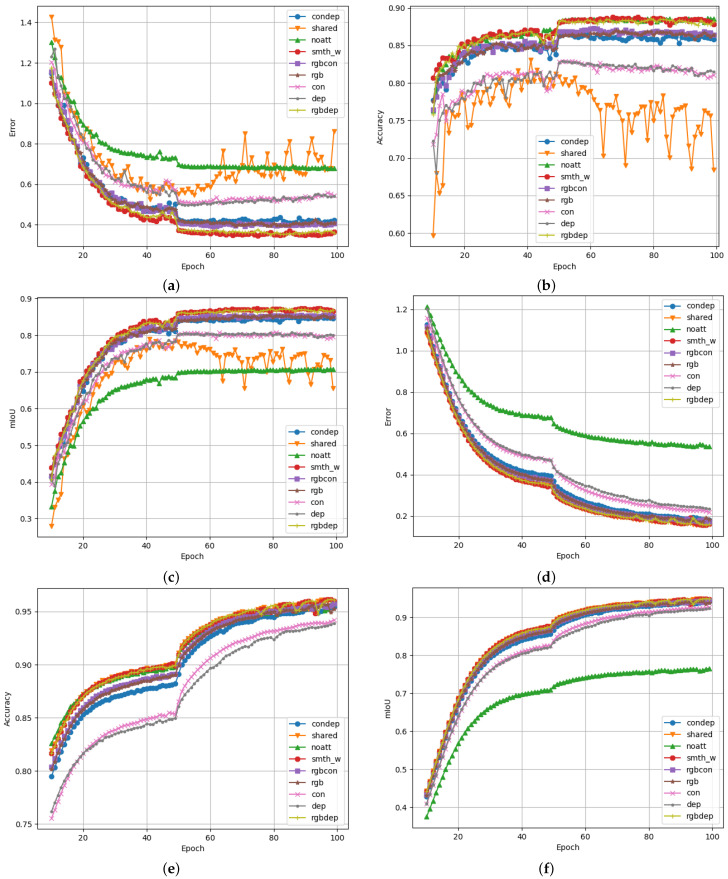
Performance graphs for the proposed scene segmentation system logged over 100 epochs of training. The total validation loss, validation accuracy, and validation mIoU are shown for Figures (**a**–**c**), respectively. Similarly, training loss, training accuracy, and training mIoU are shown for Figures (**d**–**f**), respectively. (**a**) Total validation loss as the sum of the decoder loss and the auxiliary decoder loss. (**b**) Validation accuracy calculated for each predicted pixel. (**c**) Mean intersection over union based on validation data. (**d**) Total training loss as the sum of the decoder loss and the auxiliary decoder loss. (**e**) Training accuracy calculated for each predicted pixel. (**f**) Mean intersection over union calculated over the training dataset.

Figure 10 shows the results obtained by training multiple versions of the proposed segmentation model. For each of the training iterations, a different configuration of the model was used. To evaluate the effects of adding additional data streams to the model, a training iteration was executed for every permutation of input data. In Figure 10, the line graphs labelled as “condep”, “rgbcon”, “rgbdep”, “rgb”, “con”, and “dep” represent the model configurations with no RGB data, no depth data, and no contour data; only RGB data; only contour data; and only depth data, respectively. Similarly, “shared” refers to a model configuration that uses the same encoder block for both RGB and contour input data, effectively sharing the network weights between the two input streams. This was inspired by the *s-FCN-loc* system as an efficiency improvement [27]. The “smth_w” plot refers to a data configuration where the standard proposed model was trained on 10% smoothed ground truth labels and suppressed class weighting was applied. Finally, “noatt” refers to a model configuration with no attention module blocks.

The proposed approach allows for the evaluation and comparison of these different model configurations. It is clear that some configurations performed significantly better than others in terms of accuracy and mIoU, while, at the same time, logging lower loss values, as each model was trained for more epochs. Prior experiments have shown that these training runs do not yield results than those obtained by training for more epochs. Longer training times may be beneficial for production versions of such a system but would not be useful for comparisons within the scope of this research. It should also be noted that no double-descent behaviour was observed during longer training runs. This reinforces that longer training iterations would not be very useful, likely due to the way the Adam optimiser works. Therefore, all training was set to only run for 100 epochs. The best-performing epoch would provide the weights for that specific model.

Figure 10c shows the mIoU of each training iteration, which is considered the metric that is most indicative of segmentation performance. It is clear from these training iterations that the proposed attention modules provided significant performance benefits compared to autoencoder models with no attention module. In this case, the mIoU increased from 70.8% to 87.4%.

Dual attention mechanisms offer feature aggregation of long-range dependencies in images and in the latent features of the network [50]. Since two attention mechanisms were proposed for the design of this system, spatial and channel-wise features could be analysed to correlate features that aid in road scene segmentation. The benefit of this is that the proposed system may exploit related features that are farther apart than the receptive field of the convolutional layers of the system. The specific operation of each attention module is explained in Section 3.4.

Similar improvements in mIoU were observed when comparing the smoothed and weighted model iteration with the weight-sharing iteration. The weight-sharing training iteration was the only model configuration that showed noticeable degradation of performance the longer it was allowed to train. Since this behaviour was only observed in validation data and not in training data, it can be concluded that this model configuration suffers more from overtraining/overfitting than other configurations.

A surprising was how small the performance differences were between the proposed smth_w model configuration and the rgbdep model configuration. It was clear from this result that contour data do not necessarily add that much additional information to the system, which aids it in performing effective scene segmentation on Cityscapes data. Overall, the RGB input data stream provided the most useful data to perform scene segmentation tasks. This is evident when observing that training and model configurations that include RGB data are often the top performers in these test results. This observation is expected, as the intuition behind computer vision segmentation systems is to mimic human cognition and understanding of driving scenes. Visual information is the main sense humans use to perceive dynamic environments such as these.

Table 1 presents the classification performance indicators for each of the model configurations. Precision, recall, and F1 score relate to the classification power of each model. This means that these performance indicators only convey information on how well each model can classify a pixel in an image to belong to a specific class. They do not convey information on the spatial or channel locality of the pixel being classified. This is why mIoU has been so popular in this research space, as it is directly affected by pixel locality and predicted classification. It can be observed that some models have very similar precision, recall, or F1-score values; however, their mIoU values can differ significantly for this reason. If a high number of pixels is being classified correctly but located outside the main area for that specific class, higher classification scores and lower mIoU scores may result.

Table 2 shows that the rgbdep and smth_w models similarly in some cases. The most noticeable difference is that the smth_w model performed much better between different classes than the rgbdep model. This interclass performance difference likely occurs because of the added contour image data that forms part of the smth_w model architecture. The improvements in class boundary regions allowed the proposed model to differentiate more effectively between different classes in a driving scene.

### 4.2. Dataset Configuration and Comparisons

**Figure 11:**
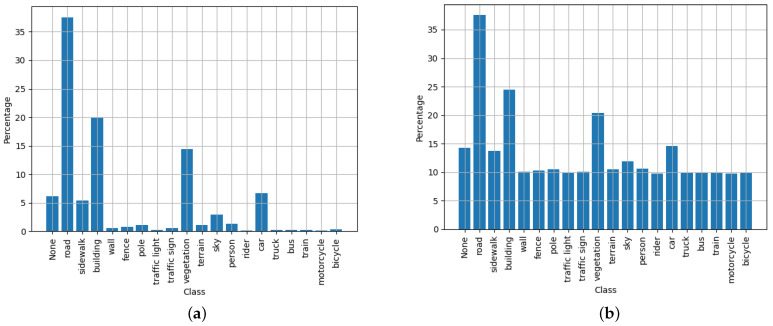
Illustration of Cityscapes normalised dataset distributions—both unweighted (**a**); and weighted (**b**) with a a tempering factor of 100 to balance out the negative training effects of under-represented classes.

For these experiments, different dataset configurations were used. It was observed that the specific configuration that yielded the best mIoU results continually required the dataset with label smoothing and class weighting applied to it. However, Figure 11 shows the distribution of each of the main classes contained in the Cityscapes dataset (20 classes). This dataset possesses up to 34 classes. However, only 20 commonly used classes are officially supported by the dataset maintainer, with more being added.

It should be noted from Figure 11 that a tempering factor of 100 was used to adjust the class weights. Through experimentation, it was observed that using the full class weights often had detrimental effects on the mIoU metric. To counter this effect, weights were limited to a maximum of 100, which prevented the class weights from equalising completely. It is hypothesised that this provides the training algorithm an opportunity to evaluate which classes have a slightly higher priority than others. This is something not as easily achieved when all classes are normalised to have the same weighting on the loss calculations.

**Figure 12:**
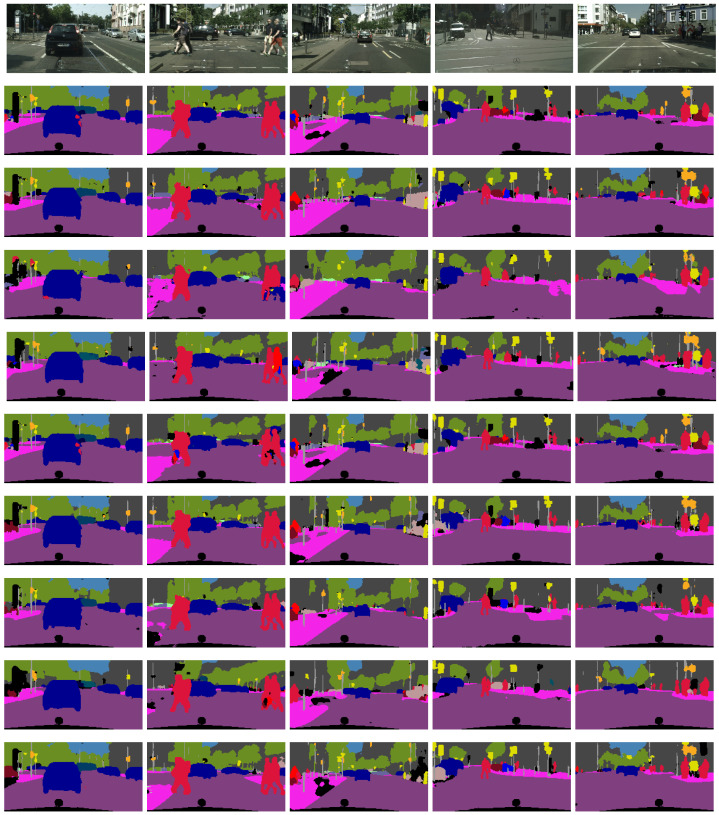
Segmentation results from each of the tested models on randomly selected validation data. The rows correlate with the order listed in Table 1, with the first row representing the original RGB images taken by the camera.

Figure 12 illustrates the segmentation results for each model listed in Table 1. The order of the segmented images correlates with the order in which they are listed in the table. The same model was used to segment each image in each row. The first row of images comprises the captured RGB images, and each image was randomly selected from the test subset.

It can be seen from Figure 12 that each model manages to perform scene segmentation of the given driving scene. The performance differences between these images are often subtle, but the details can show how robustness and consistence of each of the models. Some models struggle to keep large, solid regions that belong to the same class labelled correctly. An example of this behaviour can be seen in the third row of images in Figure 12 (shared model), as well as the second to last row (dep model). This can also be observed where the car is not fully segmented to be the same colour of pixels. Similarly, the persistence of distant traffic signs and traffic lights is a good indicator of model performance, since these objects often constitute small details that are difficult to preserve in autoencoder segmentation models.

The proposed smth_w model achieved a maximum mIoU of 87.4% during experimentation. This was higher than some of the previous mIoU scores that were achieved in other published literature that also compared the mIoU scores of their model implementations on the Cityscapes dataset. PSPNet yielded an 80.2% mIoU on the same dataset in 2017 [6]. In 2020, DSNet reported an mIoU score of 71.8% on Cityscapes data, which is lower than that achieved in this report [3]. However, DSNet did not have nearly as many trainable parameters as the model proposed in this research. It focused much more on inference efficiency rather than accurate pixel classification. Similarly, LDPNet achieved a 71.1% mIoU in 2020 [53]. Finally, looking at the benchmark section on the official Cityscapes dataset hosting website at the time of writing, several anonymous mIoU reports show that the latest research could be achieving around 85–87% mIoU. These reports are not yet verifiable and should not be taken more seriously than a rough indicator of the expected mIoU scores that modern research could achieve. It should also be noted that the resolution of the images that other systems work on can vary erratically. This could mean that a higher mIoU score does not necessarily mean that a model performs better than another model if the one with a higher score works on much lower-resolution data. Depending on how the dataset was configured, lowering the resolution typically makes the segmentation task easier for the model to perform. This was verified during experimentation.

### 4.3. CARLA Dataset Results

**Figure 13:**
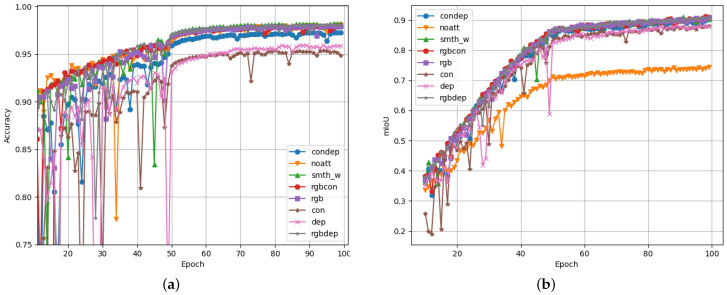
Performance graphs for the proposed scene segmentation system on the CARLA dataset logged over 100 epochs of training. The validation accuracy (**a**), and the validation mIoU (**b**) are presented as the main performance indicators.

This dataset was created using the CARLA simulator and was also used to assess the performance of the proposed scene segmentation system. The results are illustrated in Figure 13.

**Figure 14:**
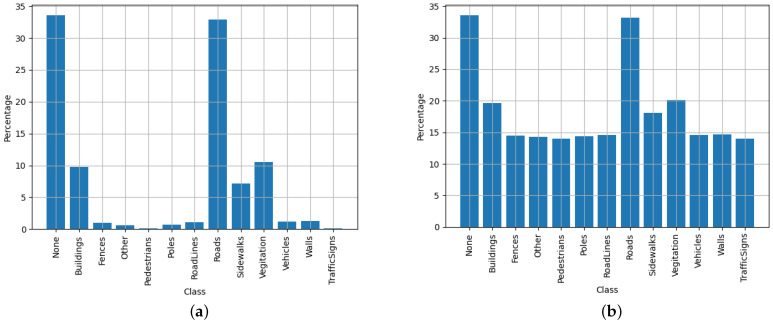
Illustration of custom CARLA-normalised dataset distributions–both unweighted (**a**) and weighted (**b**) with a tempering factor of 100 to balance out the negative training effects of under-represented classes.

The dataset distributions for the CARLA dataset are illustrated in Figure 14. This shows that the CARLA dataset only has 13 classes, which is less than the 20 tested classes from the Cityscapes dataset. Looking at the performance results in Figure 13, it is clear that the CARLA dataset yielded higher scores for accuracy and mIoU than the typical scores for the Cityscapes dataset. This was expected, as fewer classification classes typically makes the segmentation task easier to accomplish.

The evaluated segmentation performance correlates closely between the two datasets. The main observation when comparing these models on each dataset was that the CARLA dataset was significantly easier to process for each of the models. This was due to two main factors: the aforementioned lack of classification classes in the CARLA dataset and the synthetic nature of the CARLA data itself. It was noticed that the CARLA dataset was not as realistically generated as actual real-world data from the Cityscapes dataset; therefore, the segmentation effort is much less on the less realistic synthetic dataset.

## 5. Discussion

This paper proposes a more unified road scene segmentation system that strives to encourage rich semantic feature extraction from driving scenes by combining specific techniques found in related works. The main elements of the proposed system include the use of an FCN backbone model architecture, skip connections, a multi-input fusion strategy, parameter sharing, dual attention modules, and trainable upscaling. Together, this system manages to perform road scene segmentation on image data from driving scene datasets. The rest of this chapter discusses the obtained results.

### 5.1. Interpretation of Results

It was clear from the obtained results that additional input data streams had a noticeable effect on segmentation performance. This has not only been verified in previous works but also in this study. The RGB data stream was the most useful data stream that the segmentation model could process, followed by the depth image data stream and the contour image data stream. For any other permutation of the three input data streams, contour data showed the least improvement relative to a baseline model with a single-stream input architecture. Contour data seemed to only have been useful in very specific scenarios and situations, and as expected, it only focused on boundary regions between different classes. It was also observed that depth images were very useful additions to the proposed scene segmentation system, and when combined with either of the other two data types, segmentation performance increased.

Unbalanced classification classes was another factor affecting the model segmentation performance. This is especially difficult to correct when the classes contained in the datasets are very sparse. Large balancing weights can occasionally cause strange behaviour during the training process of a deep learning model. This necessitated the tempering of the balancing weights to ensure that they did not exceed a certain threshold. Significant improvement in mIoU was observed when different segmentation models were trained with class weighting applied versus when no class weighting was applied.

Label smoothing did not show system improvements overall. This was likely due to the construction of the loss function. The total loss function for this study contains terms that relate to the calculated IoU for each class. It was concluded that IoU-based loss calculations do not benefit from label smoothing in the same manner as cross-entropy loss calculations—if any benefit at all. Therefore, we recommend not applying label smoothing techniques for future iterations of the proposed scene segmentation system, instead applying class weighting strategies to compensate for class exposure bias.

Based on the obtained results, it it clear that the addition of the proposed attention module blocks resulted in significant mIoU improvements. Scene segmentation systems in the existing literature have achieved workable segmentation results; however, the addition of attention mechanisms was intended to supply a form of focus to the scene segmentation model.

It is concluded that the improvements observed in terms of the segmentation performance of the proposed model were significant enough that attention mechanisms should be included in similar future research. Depending on how these models are trained and implemented, attention blocks or modules can add significant segmentation capabilities at the cost of a larger model with more trainable parameters. This was partially verified in [50], but in this paper, we applied these attention modules in a more unified architecture alongside other segmentation strategies. This not only contributes to reaffirming the improvements to road scene segmentation performance that attention mechanisms may have but also their versatility to operate as slot-in modules in different deep learning model architectures.

### 5.2. Future Work

This research study primarily focused on road scene segmentation approaches that can be unified to improve their scene perception capabilities. We did not extensively cover other factors that influence scene segmentation performance. It was observed that model design elements such as the number of trainable parameters, network depth, and input resolution can all significantly affect segmentation performance.

Therefore, future work should focus on investigating the effects that these elements can have on more unified road scene segmentation systems. Common practice between previous studies, in terms of resolution testing, has not been extensively covered, which made it difficult to draw adequate comparisons between different system implementations. It is also known that the number of model parameters and their resolution have a direct effect on model efficiency and inference speed. How they affect factors such as classification accuracy and mIoU can still be investigated more thoroughly.

## 6. Conclusions

In this paper, we presented a novel road scene segmentation system that utilises several existing scene segmentation techniques to improve mIoU performance. This system was implemented with a more unified design than those found in related works, which allowed for rich segmentation features to be extracted from driving scenes. The focus of this paper was achieving competitive segmentation performance on well known datasets, such as the Cityscapes dataset. The proposed system achieved an mIoU score of 87.4% on the Cityscapes dataset, which is a comparable result to some of the latest reported results from the Cityscapes website [21]. Research such as that presented in this paper is currently contributing to the improvement of ADS and ADAS, thereby improving the safety of roads for all road users.

## Figures and Tables

**Table 1:** Model training configuration performance indicators. Averaged precision, recall, F1 score, and mean IoU are tabulated along with results from related works to compare mIoU scores.

Configuration	Precision	Recall	F1-Score	Support (Pixels)	Mean IoU
smth_w	0.677	0.641	0.654	25,088,000	0.874
shared	0.530	0.406	0.421	25,088,000	0.790
condep	0.614	0.563	0.578	25,088,000	0.830
rgbcon	0.617	0.575	0.587	25,088,000	0.856
rgbdep	0.691	0.619	0.642	25,088,000	0.873
rgb	0.649	0.538	0.570	25,088,000	0.856
con	0.501	0.418	0.440	25,088,000	0.808
dep	0.524	0.466	0.481	25,088,000	0.810
noatt	0.674	0.625	0.645	25,088,000	0.708
DSNet [3]	-	-	-	-	0.718
PSPNet [6]	-	-	-	-	0.802
LDPNet [53]	-	-	-	-	0.711

**Table 2:** Mean IoU calculated for each class in the Cityscapes dataset using each of the created scene segmentation models.

	smth_w	shared	condep	rgbcon	rgbdep	rgb	con	dep	noatt
None	0.461	0.315	0.459	0.435	0.500	0.423	0.373	0.418	0.416
road	0.968	0.921	0.961	0.965	0.973	0.967	0.950	0.957	0.787
sidewalk	0.786	0.598	0.703	0.762	0.723	0.738	0.625	0.722	0.545
building	0.927	0.868	0.906	0.896	0.919	0.924	0.854	0.864	0.741
wall	0.448	0.257	0.332	0.317	0.400	0.234	0.128	0.171	0.347
fence	0.396	0.097	0.360	0.275	0.350	0.184	0.169	0.195	0.322
pole	0.447	0.172	0.476	0.401	0.521	0.353	0.232	0.311	0.437
traffic light	0.427	0.077	0.397	0.283	0.481	0.213	0.002	0.170	0.401
traffic sign	0.554	0.317	0.395	0.504	0.576	0.435	0.264	0.206	0.439
vegitation	0.920	0.866	0.883	0.917	0.907	0.904	0.871	0.782	0.747
terrain	0.600	0.531	0.466	0.601	0.594	0.583	0.300	0.291	0.472
sky	0.941	0.923	0.948	0.936	0.961	0.934	0.905	0.876	0.777
person	0.741	0.640	0.706	0.657	0.782	0.747	0.558	0.593	0.618
rider	0.414	0.002	0.383	0.380	0.227	0.270	0.161	0.234	0.329
car	0.918	0.811	0.909	0.900	0.925	0.897	0.869	0.879	0.758
truck	0.472	0.006	0.284	0.414	0.530	0.380	0.036	0.380	0.327
bus	0.686	0.083	0.333	0.373	0.525	0.332	0.076	0.410	0.430
train	0.350	0.006	0.391	0.154	0.369	0.126	0.114	0.234	0.211
motor-cycle	0.267	0.002	0.104	0.344	0.105	0.234	0.008	0.010	0.144
bicycle	0.583	0.036	0.372	0.570	0.522	0.453	0.372	0.200	0.491

## Data Availability

Two datasets are references in this paper. The Cityscapes dataset is available in the Cityscapes web repository [21]. The CARLA dataset was custom-recorded from the CARLA simulator [44] and can be obtained from the first author upon request. The main training scripts that were used to create the road scene segmentation model will be made available with this paper.

## Abbreviations

The following abbreviations are used in this manuscript:

ADAS	Advanced driver assistance system
ADS	Advanced Driving system
BN	Batch normalisation
CNN	Convolutional neural network
CPU	Central processing unit
FCN	Fully convolutional network
GAN	General adversarial network
GPU	Graphical processing unit
KL	Kullback–Leibler
MDPI	Multidisciplinary Digital Publishing Institute
mIoU	Mean intersection over union
PReLU	Parametric rectified linear unit
PSU	Power supply unit
RAM	Random access memory
ReLU	Rectified linear unit
RGB	Red, green, blue
